# The preparation of calcium phosphate adsorbent from natural calcium resource and its application for copper ion removal

**DOI:** 10.1007/s11356-020-10585-7

**Published:** 2020-08-27

**Authors:** Marta Kalbarczyk, Aleksandra Szcześ, Dariusz Sternik

**Affiliations:** 1 Department of Interfacial Phenomena, Institute of Chemical Sciences, Faculty of Chemistry, Maria Curie-Skłodowska University in Lublin, Sq. M. Curie-Skłodowska 3, 20-031 Lublin, Poland; 2 Department of Physical Chemistry, Institute of Chemical Sciences, Faculty of Chemistry, Maria Curie-Skłodowska University in Lublin, Sq. M. Curie-Skłodowska 3, 20-031 Lublin, Poland

**Keywords:** Calcium phosphate, Eggshells, Wastewater, Copper ion adsorption

## Abstract

Using the hen eggshells (biowaste) as a source of calcium and an environmentally friendly approach, the nanopowder composed of 74% of hydroxyapatite (HA) and 26% of β–tricalcium phosphate (β-TCP) was obtained. Due to the maximum reduction of the stages associated with the use of chemicals and energy, this method can be considered as economically and environmentally friendly. A well-developed surface area and the negative zeta potential at pH above 3.5 indicate good adsorption properties of this material. The obtained material shows high adsorption capacity towards Cu^2+^ ions, i.e. 105.4 mg/g at pH 5. Good fit of the Langmuir adsorption model and the pseudo-second-order kinetic model may indicate chemical adsorption probably due to the electrostatic interactions between the Cu^2+^ cations and the negatively charged phosphate and hydroxyl groups on the material surface.

## Introduction

Materials based on calcium phosphates (CP) are commonly known because of their bone reconstruction possibilities, mostly in the field of dentistry, orthopaedic and trauma surgery (Jeong et al. 2019). The main difference between the mineral compositions is the Ca/P molar ratio, which can be in the range from 0.5 to 2.0 (Sakka et al. 2013). Physico-chemical properties are strongly related to the Ca/P ratio in the mineral structure; hence, the capabilities of these materials are significantly different. Applications of calcium phosphates are largely dependent on their properties. They can be used as phosphorous fertilizers or food supplements as well as the main component of cements used in dentistry according to their pH stability and solubility in water. Adaptation as bone implant materials in orthopaedic and trauma surgery is the most interesting and promising (Dorozhkin and Epple 2002; Akram et al. 2014).

The natural material, hydroxyapatite (HA), is the subject of numerous studies, among others, due to its sorption properties. This compound with chemical formula Ca_10_(PO_4_)_6_(OH)_2_ is the least soluble in water in the calcium phosphate group (pK_s_ equal to 116.8 at 25 °C) and resistant to pH changes (Akram et al. 2014). Hydroxyapatite occurs naturally in the crystalline form and, as the major constituent of vertebrate teeth and bones, is responsible for their mechanical properties. Considering its mechanical resistance and biocompatibility, scientists start to investigate its properties and synthesis methods (Jeong et al. 2019).

The HA synthesis methods can be divided into two main groups: the methods using synthetic components and those using biowaste as a source of calcium during the synthesis. From the economical and environmental points of view, the methods included in the second group are more attractive. Adaptation of such agricultural wastes as fish bones (Ratna Sunil and Jagannatham 2016), poultry bones, e.g. turkey (Esmaeilkhanian et al. 2019) and mammalian bones such as bovine or porcine (Londoño-Restrepo et al. 2019), corals (Rocha et al. 2005), sea shells (Karunakaran et al. 2019), plants (Wan et al. 2006) or eggshells (Samina and Ashan 2008; Goloshchapov et al. 2013; Sajahan et al. 2014; Bag et al. 2016; Adeogun et al. 2018; Azis et al. 2018; Tangboriboon et al. 2019) can result in a double benefit: organic waste utilization and production of attractive material with a wide list of possible applications. However, a majority of these methods require heat treatment for the decomposition of naturally occurring calcium carbonate into calcium oxide and water. The usage of a high-temperature furnace is unfavourable because of a vast amount of energy needed to create proper conditions.

There are numerous publications proving that synthetic hydroxyapatite can be effectively used as an adsorbent in wastewater and contaminated soil treatment (Bernalte et al. 2019; Panneerselvam et al. 2019; Campisi et al. 2018; Ferri et al. 2019; Das and Dhar 2020). It was found that the heavy metal removal from the solution depends on HA density, crystallinity, composition and specific surface area, as well as metal ion concentration, pH, temperature, rate of addition and ligand presence in the solution (Campisi et al. 2018; Ferri et al. 2019). Moreover, the mechanism of adsorption is connected with several phenomena like ion exchange, surface complexation and dissolution-precipitation processes responsible for creation of new, stable forms of calcium phosphates (Ferri et al. 2019).

Heavy metal contamination of industrial wastewater is still a serious problem. There are a lot of methods and technologies such as chemical precipitation, oxidation, reduction, ion exchange, reverse osmosis, ultrafiltration or electro dialysis that can be successfully used to remove heavy metal pollution from wastewater. Nevertheless, satisfying efficiency and costs is still not attained. Furthermore, these methods have inherent limitations like sensitive operating conditions, requiring several pre-treatments or generating large amounts of sludge (Renu et al. 2017). It was shown that the most effective technique for heavy metal contamination removal is adsorption using activated carbon as an adsorbent (Hegazi 2013; Nejadshafiee and Islami 2020; Song et al. 2020). The extensive use of activated carbon is not attractive from the economical point of view. Therefore, there is still a demand for searching new, cheap materials with large adsorption capacity related to heavy metal ions.

One of the most frequently prevalent metals in the environment is copper (Cu). Its trace amounts are vital for the human body due to its participation in such processes as enzyme synthesis or bones and tissue development. However, the consumption of divalent copper (Cu^2+^) is harmful because of its toxic and carcinogenic ravages. These cations accumulate in the human liver and, when the critical concentration is reached, cause a lot of symptoms like vomiting, abdominal pain, headache, nausea, liver and kidney failure, respiratory problems, gastrointestinal bleeding and consequently death (Akar et al. 2009). Not only humans but also plants and animals are in danger of being poisoned by divalent copper. The presence of the cations in the soil biota causes numerous damages to many plant species (Lamb et al. 2012). In the aquatic systems, contamination of fresh water with copper(II) leads to the animal osmo-regulatory mechanism failures (Lee et al. 2010).

The purpose of this study was to obtain a material with the highest hydroxyapatite content using raw eggshells as a source of calcium and check its adsorption capacity using copper(II) ions. The most essential assumption and the novelty element were to restrict the energy to a minimum by eliminating the stage of shell calcination and heat treatment of the precipitate and to obtain a material with the largely developed surface area.

## Materials and method

Sixty-five percent nitric acid, 96% ethyl alcohol, disodium hydrogen phosphate dodecahydrate, sodium hydroxide micropills and copper(II) chloride dihydrate were of ppa grade, and purchased from Avantor (Gliwice, Poland) and used without further purification. All solutions were prepared using water from the Milli-Q Plus system (Millipore, USA) with resistivity 18.2 MΩcm.

### Synthesis of calcium phosphate-based material

The hen eggshells were taken from the household (located in the suburbs of Lublin, Poland). The raw material was washed with distilled water and dried at 50 °C for 24 h. The dry and clean material was crumbed in a mortar to fine powder, dissolved in 1 M nitric acid and stirred for 1 h. The pH value of the solution was set at 11.40 using sodium hydroxide. The calcium content calculated from the CaCO_3_ content in the hen eggshells determined by the DTG analysis was 95% (not illustrated). Then, the stoichiometric amount (ratio Ca/P 1.67) of 0.3 M disodium hydrogen phosphate solution was added dropwise with vigorous stirring (500 rpm), with the controlled rate 20 mL/min at room temperature. The obtained mixture was stirred for 4 h followed by the ageing process at 60 °C for 24 h. The obtained material was filtered using vacuum filtration, rinsed with distilled water and 96% ethyl alcohol and dried overnight at 100 °C. The pH value of the suspension before filtration was 6.80.

### Characterization of the obtained material

The obtained mineral phase was determined by the X-ray diffraction analysis using the diffractometer Empyrean (PANalytical) and Fourier-transform infrared spectroscopy using spectrometer Nicolet IS 10 (Thermo Scientific, USA). The main idea of this method is based on the best fit of the theoretical diffractogram to the experimental one, using the least square refinements. The intensity of full diffraction pattern is used rather than a few of the highest intensities (Rietveld 1969; Scrivener et al. 2004; Feret 2008). The fitting was made using the software dedicated to the used device provided by Empyrean, PANalytical.

Morphology and crystal size of the mineral were obtained using the scanning electron microscopy (Quanta 3D FEG, FEI).

Thermal analysis was made in the atmosphere of synthetic air in the temperature range from room temperature to 1100 °C at the heating rate of 10 °C min^−1^ using a STA 449 Jupiter F1 apparatus (Netzsch, Germany) coupled with an IR spectrometer.

The specific surface area was determined by nitrogen adsorption using the Accelerated Surface Area and Porosimetry System ASAP 2420 (Micromeritics Inc., USA) and calculated using the BET equation.

The electrophoretic mobility of HA (0.01 wt%) dispersed in the 1 mM KCl solution and equilibrated for 24 h was measured by means of Zetasizer Nano (Malvern, UK). The zeta potential was calculated using the Smoluchowski equation (*U*_E_ *= ɛζ/η*, where *U*_E_ is the electrophoretic mobility, *ɛ* is the permittivity, *ζ* is the zeta potential and *η* is the viscosity of the solvent)*.*

### Adsorption studies

Adsorption studies were carried out at 25 °C using water solution of copper(II) chloride. For this purpose, 25 ± 0.2 mg of adsorbent was added to 5 mL of Cu^2+^ ion solution and the mixture was shaken at 120 rpm for a given time (from 5 to 80 min). The ion concentration was 150 mg/L and the initial pH was equal to 5. Subsequently, the supernatant was filtered with 0.22 μm PTFE hydrophilic syringe filters (AlfaChem, Poland) and the solution absorbance was measured using a spectrophotometer (Spectronic Helios Alpha Beta UV-Visible Spectrophotometer, Thermo Electron Corporation, USA) at a wavelength of 740 nm. The solution concentration was determined using the calibration curve. The amount of adsorbed ions was calculated from Eq. 1:

1 $$ a=\frac{\left({C}_0-{C}_{\mathrm{eq}}\right)\cdotp V}{m} $$

where *a* is the amount of adsorbed Cu^2+^ ions (mg/g), *C*_0_ is the initial value of copper concentration before adsorption (mg/L), *C*_eq_ is the concentration value in the supernatant in the equilibrium state (mg/L), *V* is the volume of copper solution (L) and *m* is the adsorbent mass (g).

To determine the effect of pH on the adsorption process, the pH of the solutions was adjusted in the range of 3 to 6 using 1 M NaOH or 1 M HCl solution. The initial Cu^2+^ concentration was 200 mg/L. The adsorption was made at 25 °C for 60 min.

The temperature effect was investigated at 25 °C, 35 °C and 45 °C at natural pH = 5.0 with the copper(II) ion concentration equals 200 mg/L. The adsorption time was set to 60 min.

The adsorption isotherm was recorded at an initial pH value of 5 and the copper ions initial concentrations in the range from 75 to 500 mg/L. The stable amount of adsorbent (25 mg) was added to 5 mL of solutions and stirred for 60 min (120 rpm) at 25 °C. The further procedure was as above. In order to define the character of the adsorption processes, two models were used: Langmuir and Freundlich.

The Langmuir model was expressed using the linear equation:

2 $$ \frac{C_{\mathrm{eq}}}{\ {q}_{\mathrm{eq}}}=\frac{1}{q_{\mathrm{m}}b}+\frac{C_{\mathrm{eq}}}{q_{\mathrm{m}}} $$

where *q*_eq_ and *q*_m_ are the amounts of adsorbed metal (mg/g) at the equilibrium and of monolayer respectively, *C*_eq_ is the concentration (mg/L) of metal ions in the bulk when the equilibrium is reached and *b* is the Langmuir constant (L/mg).

The Freundlich model was fitted based on the equation:

3 $$ \mathit{\ln}{q}_{\mathrm{eq}}=\frac{1}{n}\mathit{\ln}{C}_{\mathrm{eq}}+\mathit{\ln}{K}_{\mathrm{f}} $$

where *n* and *K*_f_ (mg^(1–1/*n*)^ L^1/n^ g^−1^) are the Freundlich constants with a characteristic value for a given system, *q*_eq_ is the amount of adsorbed metal (mg/g) at the equilibrium and *C*_eq_ is the equilibrium concentration (mg/L) of metal ions in the bulk.

### Adsorption kinetics

The adsorption kinetics was analysed using the pseudo-first-order and pseudo-second-order kinetic methods. The pseudo-first-order model was examined using the equation:

4 $$ \ln \left({q}_{\mathrm{e}}-{q}_{\mathrm{t}}\right)=\ln {q}_{\mathrm{e}}-{k}_1t $$

5 $$ \frac{1}{\ {q}_{\mathrm{t}}}=\frac{1}{k_2{q}_{\mathrm{e}}^2}+\frac{1}{q_{\mathrm{e}}}t $$

where *k*_1_ (min^−1^) is the velocity constant of the first-order kinetics, *k*_2_ (g/mg·min) is the velocity constant of the second-order kinetics, *q*_e_ (mg/g) is the adsorbed amount at equilibrium and *q*_t_ (mg/g) is the adsorbed amount at time *t* (min).

## Results and discussion

### Characterization of the obtained material

**Fig. 1:**
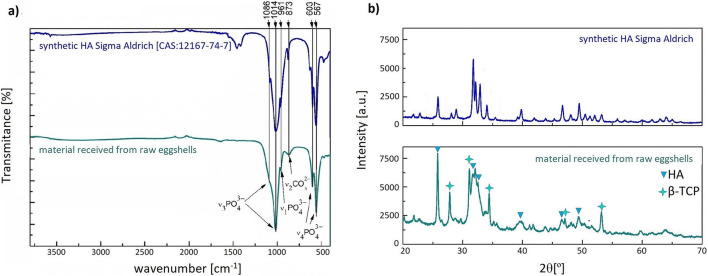
**a** FTIR spectra of synthetic HA (Sigma–Aldrich) and studied material and **b** XRD patterns of synthetic HA and studied material obtained from raw eggshells

The FTIR spectrum of the obtained mineral is presented in Fig. 1a. For comparison, the spectrum of pure synthetic HA from Sigma–Aldrich (> 97%, CAS: 12167-74-7) is also measured and included in Fig. 1a. There are shown characteristic band positions for calcium phosphates for both precipitates, such as ν_4_ PO_4_^3−^ at 567 and 603 cm^−1^, ν_2_ CO_3_^2−^at 873 cm^−1^, ν_1_ PO_4_^3−^ at 961 cm^−1^, ν_3_ PO_4_^3−^ at 1014 and 1086 cm^−1^ and ν_3_ CO_3_^2−^ at 1647 cm^−1^ (Rehman and Bonfield 1997). In the spectrum of synthetic hydroxyapatite, two additional bands at 1452 and 1422 cm^−1^ originating from ν_3_ CO_3_^2−^ situated inside the crystals lattice, as well as the peaks around 3569 cm^−1^ and 630 cm^−1^ characteristic of hydroxyl groups are presented (Panda et al. 2003; Kumta et al. 2005). The presence of signals from the carbonate vibrations is typical of natural phosphates and can be associated with the substitution of phosphate groups by the carbonate ones inside the crystals, located around 1600–1300 cm^−1^ as well as the competition between CO_3_^2−^ and the hydroxyl groups on the material surface around 873 cm ^−1^. It can be seen that the obtained synthesis product spectrum includes carbonate groups on the surface and there is no substitution of phosphate groups in the structure (Rehman and Bonfield 1997). Moreover, the analysis of peaks shape and location in the range 1100–550 cm^−1^ suggests biphasic material rather than the pure hydroxyapatite phase. The characteristic β-TCP shoulders formation in a given range and the lack of hydroxyl bands at 3569 cm^−1^ and 630 cm^−1^ confirms of dehydroxylation during the synthesis process and partial HA to β-TCP phase transformation (Kumta et al. 2005; Berzina-Cimdina and Borodajenko 2012).

The XRD method was used to characterize the crystalline form of the obtained material (Fig. 1b). For comparison, the pattern for the synthetic hydroxyapatite was also depicted. Basing on the obtained data, it can be seen that the obtained material contains two forms of calcium phosphates, i.e. hydroxyapatite and β-TCP (Sajahan et al. 2014, Tanimoto et al. 2005). The calculations based on the Rietveld’s method showed that the sample is composed of 74% of hydroxyapatite and 26% of β-TCP. Characteristic peak positions found in the obtained pattern and its comparison with the standard reference is shown in Table 1.

**Table 1:** XRD peaks positions (Tanimoto et al. 2005; Sajahan et al. 2014)

Calcium phosphate form	(h k l)	Standard reference	Synthesis product
		θ [°]	θ [°]
HA	(0 0 2)	25.9	25.8
β-TCP	(1 1 0)	25.8	
β-TCP	(2 1 4)	27.8	27.8
β-TCP	(0 2 10)	31.0	31.1
HA	(2 1 1)	31.9	31.7
HA	(1 1 2)	32.3	32.1
HA	(3 0 0)	33.0	32. 7
β-TCP	(2 2 0)	34.3	34.4
HA	(3 1 0)	39.9	39.8
HA	(2 2 2)	46.8	46.6
β-TCP	(4 0 10)	46.9	46.9
HA	(3 1 2)	48.2	48.1
HA	(2 1 3)	49.6	49.5
β-TCP	(2 2 0)	52.9	52.9

**Fig. 2:**
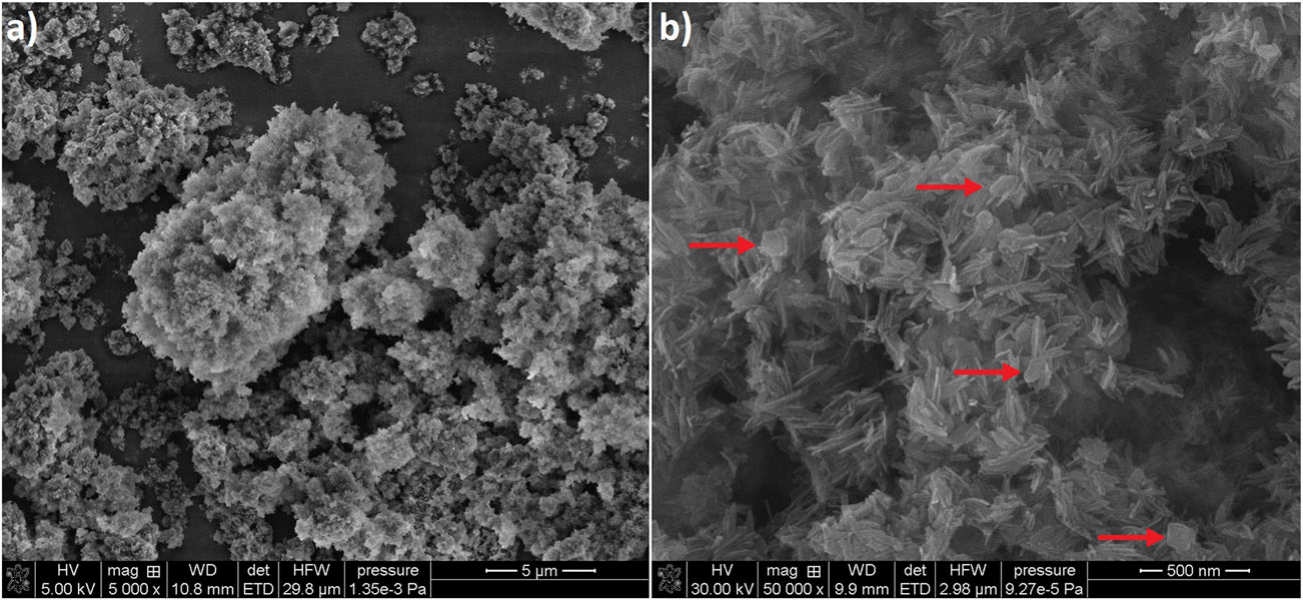
SEM pictures of the material with different magnification **a** × 5000x and **b** × 50,000

The SEM pictures of the obtained material are shown in Fig. 2. It can be seen that the material from raw eggshells occurs in the form of agglomerates with a well-developed surface. The BET surface area is equal to 66.1 m^2^/g. The material from raw eggshells is shredded. The image of higher magnification shows a mixture of characteristic rod-like structures and hexagonal-shaped plates (marked with red arrows) smaller than 250 nm.

**Fig. 3:**
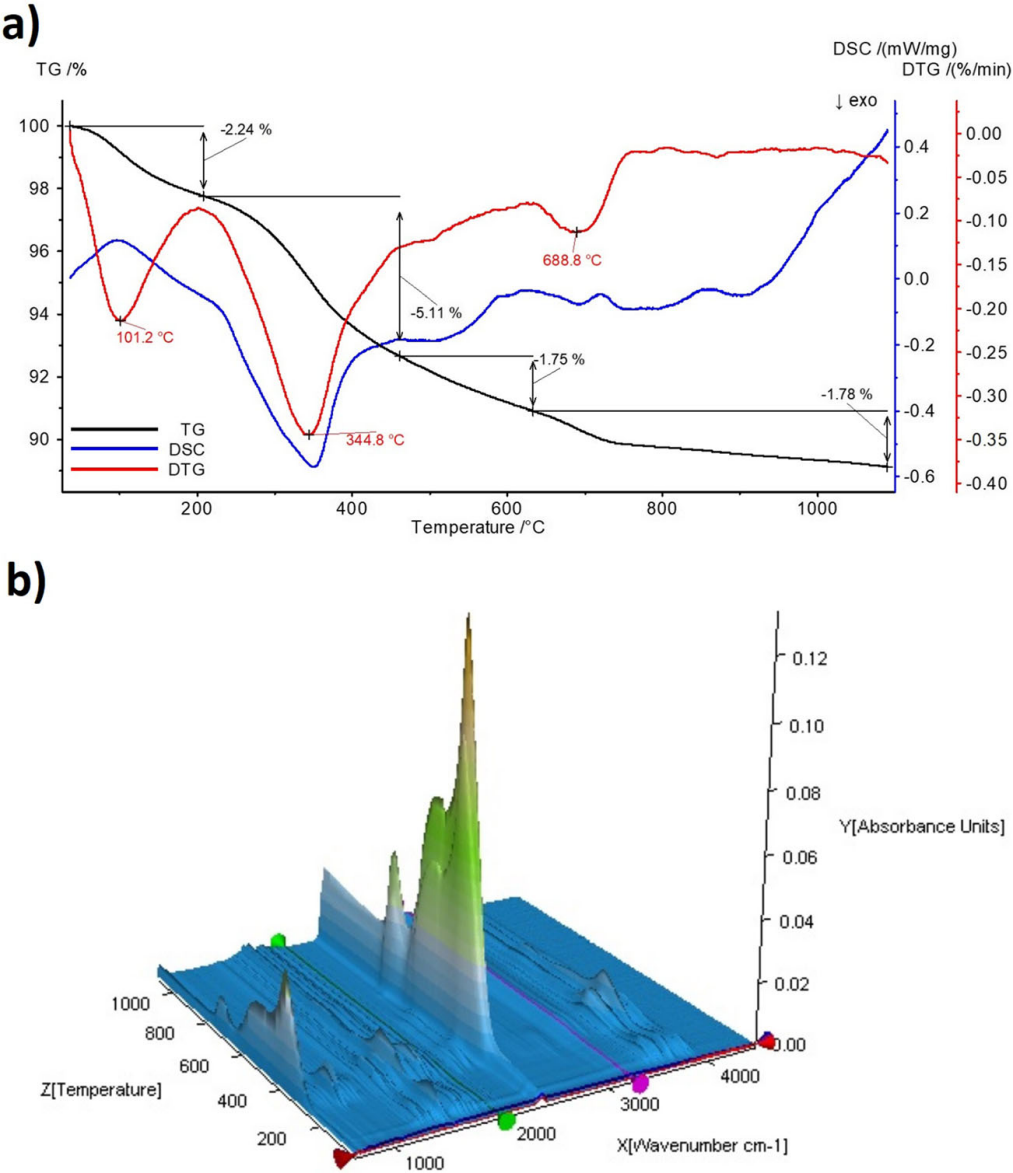
The results of the thermal analysis of the material: **a** TG, DSC, and DTG curves and **b** IR spectra of emitted gases

The thermal stability test results display three steps on the TG curve of the raw eggshell–based material corresponding to the peaks shown in the DSC and DTG dependences (Fig. 3a) with the total mass loss of 10.9%. The first one, in the temperature range up to 200 °C with the maximum at 101.2 °C on the DTG curve, is associated with water loss (2.24% of the total sample mass). The loss of water is accompanied by the endothermic peaks on the DSC curve around 100 °C.

Another weight loss in the temperature range 200–450 °C with maximum at 344.8 °C was observed. This weight loss equal to 5.11% may originate from the degradation of organic remains and lattice water. The peak at 344.8 °C in the DTG curve corresponds to the exothermic peak at the same temperature in the DSC curve. The latter change was in the range from 450 to 1100 °C. The gradual change from 450 to 1100 °C is probably connected with the slow process of elimination of carbonate groups and disposal of water released from the hydroxyapatite particles during the transformation to the more thermal stable form β-TCP compatible with the endothermic peaks shown on the DSC curve (Kumta et al. 2005; Meejoo et al. 2006). Elimination of water and carbon dioxide originating from the carbonate groups is confirmed by the IR spectrum registered during the thermal decomposition process (Fig. 3b). At the temperature around 100 °C, peaks of an intensity around 3500 cm^−1^ corresponding to a water loss are visible. The peaks in the same location, but with greater intensity in the temperature range 200–450 °C, confirm the loss of lattice water. IR vibrations of carbon dioxide at ca. 2400 cm^−1^ and 550 cm^−1^ in the temperature range 200–450 °C and 450–1100 °C confirm its elimination as a result of decomposition of organic residues and carbonate groups during the HA transformation.

### Adsorption and adsorption kinetics studies

**Fig. 4:**
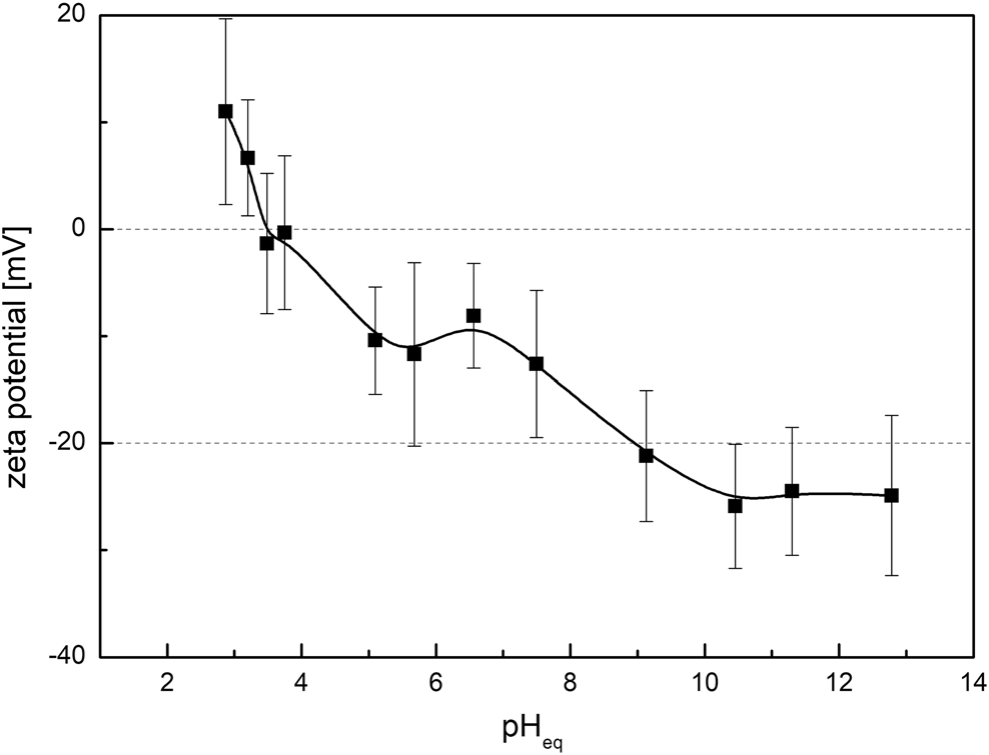
Zeta potential (mV) as a function of equilibrium pH

The zeta potential changes vs. the equilibrium pH dependence (Fig. 4) can be used to define the charge of particles in the aquatic system. The isoelectric point of the obtained material is around pH 3.5. Above this pH, the zeta potential was negative indicating that the surface is negatively charged. Low value of the isoelectric point (IEP) corresponding to the zero zeta potential provides the dominance of anionic group on the material surface which can be connected with the presence of hydroxyl and phosphate groups. In the alkaline medium, the value of zeta potential oscillates around − 25 mV and is constant. The presence of negatively charged surface groups may facilitate the adsorption of positively charged ions.

**Fig. 5:**
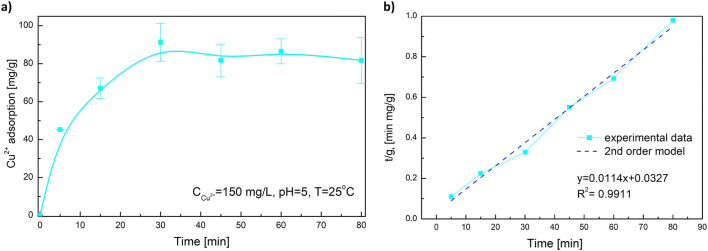
**a** Time effect on Cu^2+^ adsorption, **b** pseudo-second-order adsorption kinetics

The time impact on the amount of adsorbed copper ions on the studied material surface is presented in Fig. 5a. It can be noticed that the adsorption rate for copper ions is fast and the adsorption equilibrium in the system was reached after 30 min. After that time, the average value of adsorbed copper ions remained at 86 mg/g.

The adsorption kinetics was studied using the pseudo-first-order and pseudo-second-order kinetic methods. The correlation factor (*R*^2^) for the pseudo-first-order model was very low and equal to 0.718 (data not given). The good fitting of the pseudo-second-order kinetics (Fig. 5b) may indicate that the cooper ion adsorption on obtained materials is rather chemisorption than physisorption (Núñez et al. 2019). The calculated values of *q*_e_ and *k*_2_ were 87.7 mg/g and 0.004 g·mg^−1^·min^−1^, respectively.

**Fig. 6:**
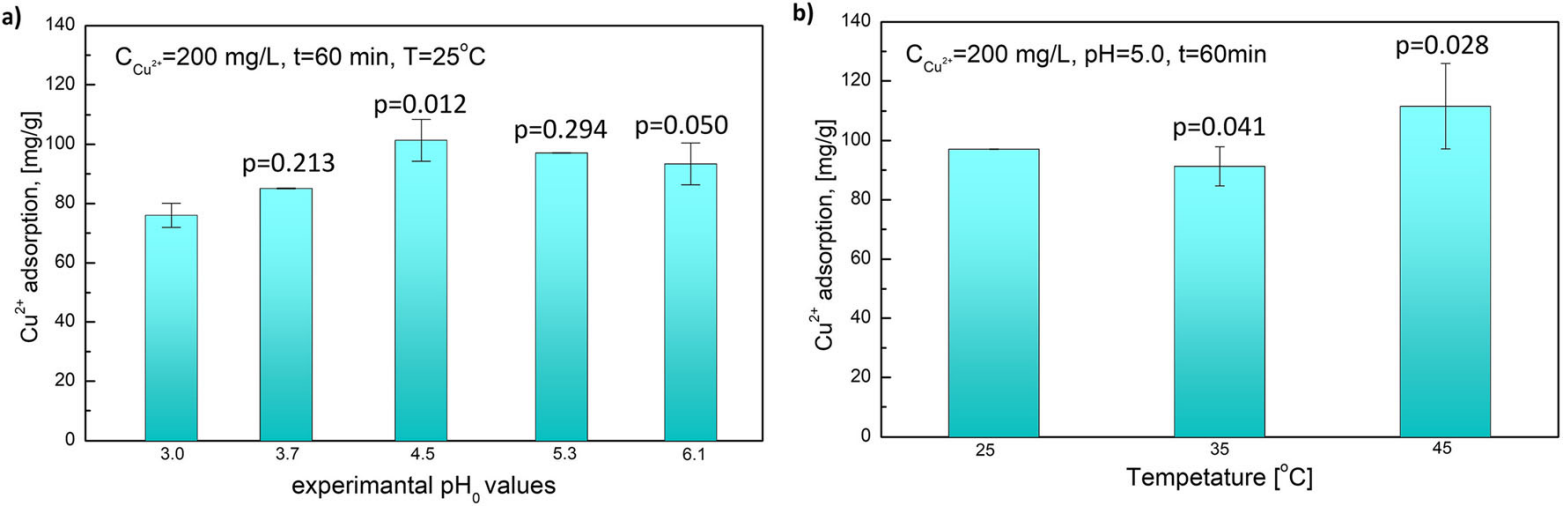
pH (**a**) and temperature (**b**) effect on Cu^2+^ adsorption. The analysis of probabilities is listed above each column

Figure 6 a shows the impact of solution pH on the copper ion adsorption. As can be seen, the pH value plays meaningful role in copper ion adsorption. In the pH range from 3 to 4.5, the adsorption increases from 76.0 mg/g to its maximum value 101.4 mg/g. When the acidity of the solution was decreasing from 4.5 to 6.0, the downward trend was observed. Copper hydroxide precipitation was responsible for the value differences of measurement results at high pH values. The impact of temperature on the copper ion adsorption is shown in Fig. 6b. The changes of adsorption capacity due to a temperature increase do not present a regular trend. At 25 °C, Cu^2+^ adsorption was equal to 97.1 mg/g of adsorbent. After increasing the temperature to 35 °C, the adsorption efficiency decreased slightly, but within the standard deviation, and reached the value of 91.3 mg/g. Further temperature change to 45 °C resulted in a further increase in the adsorption capacity to 111.6 mg/g.

The impact of pH and temperature was evaluated statistically using unpaired Student’s *t* test (Fig. 6). It can be seen than in addition to pH 3.7, all results are statistically significant, i.e. *p* < 0.05

**Fig. 7:**
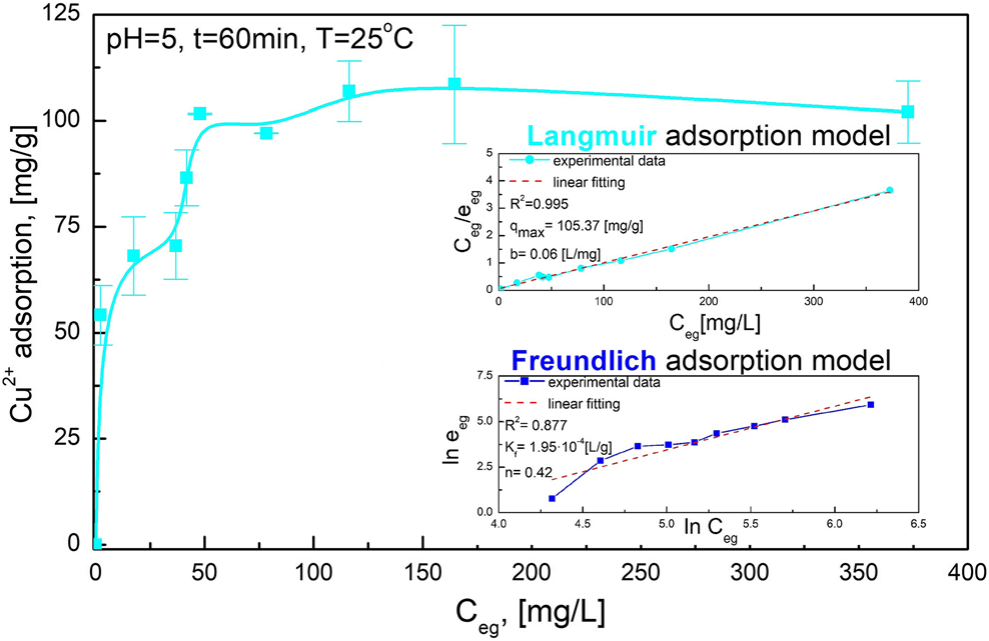
Adsorption isotherm for copper ions (**a**) and Langmuir (**b**) and Freundlich (**c**) model fitting

The adsorption isotherm is shown in Fig. 7a. The pH value of the system was chosen in order to avoid copper hydroxide precipitation. The curve shape can be described using the well-established adsorption models such as Langmuir and Freundlich.

The Langmuir equation is a two-parameter model: the first refers to the amount of Cu^2+^ ions adsorbed per unit of adsorbent mass consistent with the complete monolayer coverage *q*_m_ and the second is the Langmuir constant connected with the free energy of adsorption *K*_L_ (Hsieh and Teng 2000). It can be noticed that the dependence of the *C*_eg_/*e*_eg_ ratio and *C*_eg_ gives a linear plot with the very high value of coefficient *R*^2^ = 0.995. According to the obtained data, the complete monolayer coverage *q*_m_ and the Langmuir constant *K*_L_ are equal to 105.4 mg/g and 0.06 L/mg, respectively (Fig. 7b).

The Freundlich adsorption model refers to heterogeneous and microporous surface and introduces such parameters as the constants related to the adsorption capacity *K*_F_ and the heterogeneity factor *n*. The calculations gave such values as 1.95 × 10^−4^ L/g for *K*_F_ and 0.42 for *n*. This plot can not be fitted as a linear one with satisfying results; the value of coefficient *R*^2^ is 0.877 (Fig. 7c). The comparison of the two models allows stating that better results were obtained for Langmuir model.

As can be seen in Table 2, the obtained adsorption capacity *q*_m_ is higher than that for the other mentioned HA materials (Ansari et al. 2019; Orlova et al. 2019; Núñez et al. 2019) and over 4 times higher than for the modified activated carbons obtained by Gu et al. (2019). This indicates that the obtained HA-based material is a promising adsorbent which will be confirmed by further studies using other heavy metals ions.

**Table 2:** Comparison of the obtained adsorption parameters with the literature reports

Used adsorbent	Langmuir model			Conditions	Ref.
	*q*_m_ (mg/g)	*K*_L_ (L/mg)	*R*^2^		
Modified activated carbons	23.9	0.003	0.971	pH = 5, T = 30 °C	Gu et al. 2019
Fe_3_O_4_/hydroxyapatite/β-cyclodextrin nanocomposite	66.7	0.1972	0.997	pH = 6, T = 25 °C	Ansari et al. 2019
Hydroxyapatite	81.6	0.00012	0.920	Neutral pH, T = 25 °C	Orlova et al. 2019
Hydroxyapatite	55.9	0.060	0.99	pH = 5, T = 25 °C	Núñez et al., 2019
Hydroxyapatite-based material	105.4	0.06	0.995	pH = 5, T = 25 °C	This study

## Conclusions

In this study, the agricultural waste in the form of chicken eggshells and disodium hydrogen phosphate solution was used to obtain calcium phosphate nanomaterial via the wet precipitation method. The material composed of 74% of hydroxyapatite and 26% of tricalcium phosphate was synthesized while limiting consumption of chemicals and energy. This powder exhibited the well-developed surface area and the negative zeta potential above pH equal 3.5. The adsorption capacity of copper(II) ions on the obtained material was equal to 105.4 mg/g at pH 5. The highest adsorption was found at pH 4.5 and temperature 45 °C. A better fit of the Langmuir adsorption model than the Freundlich one and the pseudo-second-order kinetic model may indicate chemical adsorption which requires further confirmation by the thermodynamic study. Strong adsorption of copper cations is most likely a result of their interactions with the negatively charged surface of the tested material.

The powder composition that differs in solubility may indicate its potential use as biomaterial in implant production, but this requires further research.

## References

[CR1] Adeogun IA, Ofudje AE, Idowu MA (2018). Facile development of nano size calcium hydroxyapatite based ceramic from eggshells: synthesis and characterization. Waste Biomass Valor.

[CR2] Akar ST, Akar T, Kaynak Z, Anilan B, Cabuk A, Tabak ÃZ, Demir TA, Gedikbey T (2009). Removal of copper(II) ions from synthetic solution and real wastewater by the combined action of dried Trametes versicolor cells and montmorillonite. Hydrometallurgy.

[CR3] Akram M, Ahmed R, Shakir I, Wan Ibrahim WA, Hussain R (2014). Extracting hydroxyapatite and its precursors from natural resources. J Mater Sci.

[CR4] Ansari A, Vahedi S, Tavakoli O, Khoobi M, Faramarzi MA (2019). Novel Fe3O4/hydroxyapatite/β-cyclodextrin nanocomposite adsorbent: synthesis and application in heavy metal removal from aqueous solution. Appl Organometal Chem.

[CR5] Azis Y, Adrian M, Alfarisi CD, Khairat R, Sri M (2018). Synthesis of hydroxyapatite nanoparticles from egg shells by sol-gel method. OP Conf Ser: Mater Sci Eng.

[CR6] Bag S, Ganguly K, Biswas BK (2016). Structural characterization of nanocrystalline hydroxyapatite produced from waste egg shell, trends biomater. Artif Organs.

[CR7] Bernalte E, Kamieniak J, Randviir EP, Bernalte-Garcia A, Banks CE (2019). The preparation of hydroxyapatite from unrefined calcite residues and its application for lead removal from aqueous solutions. RSC Adv.

[CR8] Berzina-Cimdina L, Borodajenko N (2012) Research of calcium phosphates using Fourier transform infrared spectroscopy. Infrared Spectroscopy- Materials Science, Engineering and Technology, Theophile Theophanides, IntechOpen, 123–148

[CR9] Campisi S, Castellano C, Gervasini A (2018). Tailoring the structural and morphological properties of hydroxyapatite materials to enhance the capture efficiency towards copper(II) and lead(II) ions. New J Chem.

[CR10] Das KC, Dhar SS (2020) Removal of cadmium(II) from aqueous solution by hydroxyapatite-encapsulated zinc ferrite (HAP/ZnFe_2_O_4_) nanocomposite: kinetics and isotherm study. Environ Sci Pollut Res. 10.1007/s11356-020-09832-8 10.1007/s11356-020-09832-8 32613515

[CR11] Dorozhkin SV, Epple M (2002). Biological and medical significance of calcium phosphates. Angew Chem Int Ed.

[CR12] Esmaeilkhanian A, Sharifianjazi F, Abouchenari A, Rouhani A, Parvin N, Irani M (2019). Synthesis and characterization of natural nano-hydroxyapatite derived from Turkey femur-bone waste. Appl Biochem Biotechnol.

[CR13] Feret FR (2008) Breakthrough in analysis of electrolytic bath using Rietveld-XRD method in light metals. Edited by De Young D.H, The Minerals, Metals and materials Society

[CR14] Ferri M, Campisi S, Scavini M, Evangelisti C, Carniti P, Gervasini A (2019). In-depth study of the mechanism of heavy metal trapping on the surface of hydroxyapatite. Appl Surf Sci.

[CR15] Goloshchapov DL, Kashkarov VM, Rumyantseva NA, Seredin PV, Lenshin AS, Agapov BL, Domashevskaya EP (2013). Synthesis of nanocrystalline hydroxyapatite by precipitation using hen’s eggshell. Ceram Int.

[CR16] Gu SY, Hsieh CT, Gandomi YA, Yang ZF, Li L, Fu CC, Juang RS (2019). Functionalization of activated carbons with magnetic iron oxide nanoparticles for removal of copper ions from aqueous solution. J Mol Liq.

[CR17] Hegazi HA (2013). Removal of heavy metals from wastewater using agricultural and industrial wastes as adsorbents. HBRC J.

[CR18] Hsieh C-T, Teng H (2000). Influence of mesopore volume and adsorbate size on adsorption capacities of activated carbons in aqueous solutions. Carbon.

[CR19] Jeong J, Kim JH, Shim JH, Hwang NS, Heo CY (2019). Bioactive calcium phosphate materials and applications in bone- regeneration. Biomater Res.

[CR20] Karunakaran G, Cho E-B, Kumar GS, Kolesnikov E, Janarthanan G, Pillai MM, Rajendran S, Boobalan S, Gorshenkov MV, Kuznetsov D (2019). Ascorbic acid-assisted microwave synthesis of mesoporous Ag-doped hydroxyapatite nanorods from biowaste seashells for implant applications. ACS Appl Bio Mater.

[CR21] Kumta PN, Sfeir C, Lee D-H, Olton D, Choi D (2005). Nanostructured calcium phosphates for biomedical applications: novel synthesis and characterization. Acta Biomater.

[CR22] Lamb DT, Naidu R, Ming H, Megharaj M (2012). Copper phytotoxicity in native and agronomical plant species. Ecotoxicol Environ Saf.

[CR23] Lee JA, Marsden ID, Glover CN (2010). The influence of salinity on copper accumulation and its toxic effects in estuarine animals with differing osmoregulatory strategies. Aquat Toxicol.

[CR24] Londoño-Restrepo SM, Jeronimo-Cruz R, Millán-Malo BM, Rivera-Muñoz EM, Rodriguez-García EM (2019). Effect of the nano crystal size on the X-ray diffraction patterns of biogenic hydroxyapatite from human, bovine, and porcine bones. Sci Rep.

[CR25] Meejoo S, Maneeprakorn W, Winotai P (2006). Phase and thermal stability of nanocrystalline hydroxyapatite prepared via microwave heating. Thermochim Acta.

[CR26] Nejadshafiee V, Islami MR (2020). Intelligent-activated carbon prepared from pistachio shells precursor for effective adsorption of heavy metals from industrial waste of copper mine. Environ Sci Pollut Res.

[CR27] Núñez D, Serrano JA, Mancisidor A, Elgueta E, Varaprasad K, Oyarzun P, Caceres R, Ide W, Rivas BL (2019). Heavy metal remowal from aqueous systems using hydroxyapatite nanocrystals derived from clam shells. RSC Adv.

[CR28] Orlova MA, Nikolaev AL, Trofimova TP, Severin AV, Gopin AV, Zolotova NS, Dolgova VK, Orlov AP (2019). Specific properties of hydroxyapatite as a potential transporter of copper ions and its complexes. Russ Chem Bull Int Ed.

[CR29] Panda RN, Hsieh MF, Chung RJ, Chin TS (2003). FTIR, XRD, SEM and solid state NMR investigations of carbonate-containing hydroxyapatite nano-particles synthesized by hydroxide-gel technique. J Phys Chem Solids.

[CR30] Panneerselvam K, Thanigai AK, Warrier AR, Asokan K, Dong CL (2019). Rapid adsorption of industrial pollutants using metal ion doped hydroxyapatite. AIP Conf Proceed.

[CR31] Rehman I, Bonfield W (1997). Characterization of hydroxyapatite and carbonated apatite by photo acoustic FTIR spectroscopy. J Mater Sci.

[CR32] Renu MA, Singh K, Upadhyaya S, Dohare RK (2017). Removal of heavy metals from wastewater using modified agricultural adsorbents. Mater Today: Proc.

[CR33] Rietveld HMA (1969). A profile refinement method for nuclear and magnetic structures. J Appl Crystallogr.

[CR34] Rocha JHG, Lemos AF, Agathopoulos S, Valerio P, Kannan S, Oktar FN, Ferreira JMF (2005). Scaffolds for bone restoration from cuttlefish. Bone.

[CR35] Sajahan NA, Wan W, Mohd A (2014). Microwave irradiation of nano-hydroxyapatite from chicken eggshells and duck eggshells. Sci World J.

[CR36] Sakka S, Bouaziz J, Ayed FB (2013) Mechanical properties of biomaterials based on calcium phosphate and bioinert oxides for applications. Advances in biomaterials science and biomedical applications in Biomedicine. Intech, Rijeka, 23–50

[CR37] Samina A, Ahsan M (2008). Synthesis of Ca-hydroxyapatite bioceramic from egg shell and its characterization. J Sci Ind Res.

[CR38] Scrivener KL, Füllmann T, Gallucci E, Walenta G, Bermejo E (2004). Quantitative study of Portland cement hydration by X-ray diffraction/Rietveld analysis and independent methods. Cem Concr Res.

[CR39] Song Y, Wang L, Lv B, Chang G, Jiao W, Liu Y (2020). Removal of trace Cr(VI) from aqueous solution by porous activated carbon balls supported by nanoscale zero-valent iron composites. Environ Sci Pollut Res.

[CR40] Sunil RB, Jagannatham M (2016). Producing hydroxyapatite from fish bones by heat treatment. Mat Lett.

[CR41] Tangboriboon N, Suttiprapar J, Changkhamchom S, Sirivat A (2019) Alternative green preparation of mesoporous calcium hydroxyapatite by chemical reaction of eggshell and phosphoric acid. Int J Appl Ceram Technol 1-9

[CR42] Tanimoto Y, Hayakawa T, Nemoto K (2005). Tape-casting technique can prepare β-TCP sheets with uniform thickness and flexibility. J Biomed Mater Res Part B: Appl Biomater.

[CR43] Wan YZ, Hong L, Jia SR, Huang Y, Zhu Y, Wang YL, Jiang HJ (2006). Synthesis and characterization of hydroxyapatite–bacterial cellulose nanocomposites. Compos Sci Technol.

