# Correction to: The preparation of calcium phosphate adsorbent from natural calcium resource and its application for copper ion removal

**DOI:** 10.1007/s11356-023-25627-z

**Published:** 2023-02-17

**Authors:** Marta Kalbarczyk, Aleksandra Szcześ, Dariusz Sternik

**Affiliations:** 1 Department of Interfacial Phenomena, Institute of Chemical Sciences, Faculty of Chemistry, Maria Curie-Skłodowska University in Lublin, Sq. M. Curie-Skłodowska 3, 20-031 Lublin, Poland; 2 Department of Physical Chemistry, Institute of Chemical Sciences, Faculty of Chemistry, Maria Curie-Skłodowska University in Lublin, Sq. M. Curie-Skłodowska 3, 20-031 Lublin, Poland

**Correction to: Environmental Science and Pollution Research (2021) 28:1725–1733**

10.1007/s11356-020-10585-7

While reviewing our article we found that in the section “Synthesis of calcium phosphate-based material” the stoichiometric ratio Ca/P was given 1.67 while the actual ratio was 0.6. This corrigendum does not affect the discussion and conclusions of the original paper because the obtained material was characterized correctly. We would like to apologize for any inconvenience caused.

